# Lipedema and Dynapenia: Inflammatory Myosteatosis as a Mechanistic Link Between Tissue Expansion and Muscle Dysfunction

**DOI:** 10.3390/ijms27052319

**Published:** 2026-03-01

**Authors:** Diogo Pinto da Costa Viana, Adriana Luckow Invitti, Eduardo Schor

**Affiliations:** 1 Department of Gynecology, Escola Paulista de Medicina, Federal University of Sao Paulo (EPM-UNIFESP), Sao Paulo 04024-002, Brazil; 2 Brazilian Society for Research and Teaching in Medicine (SOBRAPEM), Sao Paulo 01318-901, Brazil; 3 Brazilian Society of Obesity Medicine (SBEMO), Florianópolis 88070-800, Brazil

**Keywords:** lipedema, dynapenia, myosteatosis, skeletal muscle dysfunction, mitochondrial diseases, tirzepatide, adipose tissue, skeletal muscle

## Abstract

Lipedema is a chronic, progressive adipose tissue disorder characterized by disproportionate subcutaneous fat accumulation, pain, edema, and resistance to conventional weight-loss strategies. Although traditionally conceptualized as a disease of adipose expansion, increasing clinical and imaging evidence suggests that functional impairment in advanced lipedema cannot be explained by adipose pathology alone. This narrative, hypothesis-generating review proposes an integrated pathophysiological framework in which inflammatory myosteatosis serves as a mechanistic bridge between lipedema progression and dynapenia. We examine how chronic adipose inflammation, microvascular dysfunction, and impaired lipid mobilization may promote ectopic lipid deposition within skeletal muscle, leading to mitochondrial inflexibility, oxidative stress, and reduced contractile efficiency. Within this model, lipedematous dynapenic myosteatosis may explain the paradox of reduced muscle strength despite preserved or increased limb volume, particularly during the transition from Stage 2.5 to Stage 3. Unlike obesity-associated dynapenia, which is primarily driven by systemic metabolic overload, lipedema-related muscle dysfunction may involve localized adipose–muscle inflammatory crosstalk and mechanical intolerance to exercise. This framework reframes advanced lipedema as a disorder of coupled adipose–muscle dysfunction rather than isolated adipose excess. The model is conceptual and intended to generate testable hypotheses, highlighting the need for future studies incorporating objective measures of muscle quality, mitochondrial function, and inflammatory signaling to clarify mechanisms underlying functional decline.

## 1. Introduction

Lipedema is a chronic, progressive adipose tissue disorder characterized by symmetrical and disproportionate accumulation of subcutaneous adipose tissue (SAT), predominantly affecting the lower extremities and, frequently, the arms while sparing the hands and feet [1,2,3]. First described by Allen and Hines in 1940, lipedema affects almost exclusively women, with prevalence estimates ranging from 6% to 11% of the female population [1,2,3]. This marked female predominance strongly suggests a hormonal component, supported by the frequent onset or exacerbation of symptoms during periods of endocrine transition such as puberty, pregnancy, and menopause [4,5,6]. Estrogen receptor imbalance, altered adipose steroid metabolism, and sex-specific fat distribution patterns have been proposed as contributing mechanisms underlying this sex bias [4,5].

Beyond female sex, several additional risk factors have been associated with lipedema, including positive family history, hormonal fluctuations, obesity, and conditions characterized by chronic low-grade inflammation [4,7]. Although obesity is not synonymous with lipedema, excess adiposity may exacerbate mechanical load, inflammatory signaling, and lymphatic dysfunction, thereby accelerating disease progression in susceptible individuals. Genetic predisposition has also been suggested, as familial clustering is frequently reported, although specific causative genes remain incompletely defined. Although sex hormones likely contribute to disease susceptibility, the present review focuses primarily on adipose–muscle interactions and the mechanisms underlying functional decline in advanced stages.

Clinically, lipedema is distinguished from common obesity by orthostatic edema, capillary fragility with easy bruising, spontaneous pain, and resistance to fat mobilization despite caloric restriction [4,8]. However, despite its distinctive phenotype, lipedema remains underrecognized and is often misclassified as lymphoedema, gynoid obesity, or simple deconditioning. This diagnostic ambiguity contributes to prolonged delays—often exceeding a decade—during which patients are frequently advised to intensify caloric restriction and physical activity with limited clinical benefit [9,10,11].

Recent disease re-characterization by Al-Ghadban et al. introduced a refined staging system (Stages 1, 1.5, 2, 2.5, and 3) that integrates morphological progression with emerging functional considerations [12]. While early stages are characterized predominantly by adipose tissue expansion and nodularity, Stage 3 demonstrates marked tissue deformation, increased inflammatory burden, and impaired mobility performance. Notably, skeletal muscle mass increases in parallel with fat mass in advanced stages; however, this expansion may not reflect true functional hypertrophy. Stage 3 is associated with impaired mobility performance and a dissociation between skeletal muscle mass and functional capacity, findings that are consistent with a dynapenic phenotype rather than true hypertrophic adaptation. Importantly, Stage 2.5 has been proposed as a transitional phenotype, in which early functional decline may emerge before overt disability becomes clinically evident. This transitional stage provides a critical window for investigating mechanisms underlying functional deterioration. For clarity, staging in the present framework follows the classification proposed by Al-Ghadban et al., including Stage 2.5 as a transitional morpho-functional phenotype. Dynapenia is operationally assessed using established consensus thresholds for reduced muscle strength, such as validated cut-offs for handgrip strength and performance-based measures (e.g., TUG and STS). Although lipedema-specific cut-offs have not yet been formally defined, the application of standardized strength criteria allows objective stratification of functional impairment across stages.

One emerging but insufficiently explored dimension of lipedema progression is skeletal muscle dysfunction. In contrast to classical obesity, where increased body mass is often accompanied by compensatory increases in muscle mass, women with advanced lipedema frequently exhibit reduced muscle strength relative to limb volume [13,14]. This dissociation is consistent with dynapenia, defined as the age- or disease-related loss of muscle strength that is not fully explained by loss of muscle mass alone [15].

Dynapenia is a multifactorial condition involving impaired neuromuscular activation, alterations in muscle fiber composition (particularly type II fiber atrophy), mitochondrial dysfunction, chronic inflammation, insulin resistance, reduced protein synthesis, and physical inactivity. Chronic low-grade inflammation has been associated with reduced muscle strength and functional decline [16]. Unlike sarcopenia, which primarily emphasizes quantitative muscle loss, dynapenia reflects deterioration in muscle quality and contractile efficiency. Intramuscular fat accumulation has been shown to impair muscle quality and functional performance [17], while physical inactivity and short-term disuse can rapidly induce measurable declines in muscle strength and metabolic function [18].

We hypothesize that lipedema may influence several of these dynapenic pathways. Chronic adipose inflammation, microvascular dysfunction, interstitial fluid accumulation, and impaired lipid mobilization may promote ectopic lipid deposition within skeletal muscle, leading to inflammatory myosteatosis. This process may compromise mitochondrial fatty acid oxidation, increase reactive oxygen species production, and impair excitation–contraction coupling, ultimately reducing muscle strength despite preserved or even increased limb volume. In advanced stages, particularly Stage 3, this phenomenon may manifest clinically as a dynapenia-like functional phenotype.

A critical therapeutic paradox emerges at this stage. Resistance training is the established first-line strategy to improve muscle strength and prevent dynapenia in most populations. However, in lipedema, especially in transitional Stage 2.5 and Stage 3, high-intensity mechanical loading may exacerbate inflammation, pain, and edema, potentially limiting exercise tolerance. This creates a physiological deadlock in which the primary intervention for dynapenia may be poorly tolerated in the very condition that requires it.

The objective of this narrative review is not merely to summarize existing evidence but to propose a mechanistically integrated framework linking lipedema progression, inflammatory myosteatosis, and dynapenia within the context of the updated staging system. By focusing on the transition from Stage 2.5 to Stage 3, we aim to clarify how adipose–muscle crosstalk may contribute to functional decline and to generate testable hypotheses regarding potential metabolic and contractile modulation strategies. This framework is presented as hypothesis-generating and translational, intended to stimulate future experimental and clinical investigation rather than to establish therapeutic recommendations.

## 2. The Clinical Paradox: Lipedematous Dynapenia

The staging framework referenced throughout this section follows the refined classification proposed by Al-Ghadban et al., which includes Stages 1, 1.5, 2, 2.5, and 3. While morphological progression occurs across the entire spectrum, functional dissociation between tissue volume and muscle performance becomes most evident in advanced stages. The present analysis acknowledges all stages but focuses mechanistically on the transition from Stage 2.5 to Stage 3 as the critical window of functional inflection.

### 2.1. The Illusion of Mass: Pseudo-Hypertrophy Versus Functional Reality

In classical obesity, the relationship between body mass and muscle strength is often approximately linear, as increased gravitational load requires compensatory hypertrophy of antigravity muscles to preserve locomotion. This adaptation is largely physiological and functionally meaningful. Lipedema, however, represents a marked deviation from this allometric pattern.

In early stages (Stages 1 and 1.5), adipose expansion and textural changes predominate, with limited objective evidence of functional compromise. Stage 2 is characterized by nodular progression and increasing tissue heterogeneity. Stage 2.5 represents a transitional morpho-functional phenotype marked by early lobular formation and emerging mechanical restriction. It is within this transitional phase that subtle dissociation between limb volume and contractile performance may begin to manifest, preceding overt functional impairment recognized in Stage 3.

Clinical examination of patients in Stages 2.5 and 3 frequently reveals lower limbs with substantial volume which, under superficial assessment or bioelectrical impedance analysis (BIA), may be interpreted as reflecting increased fat-free mass (FFM). This apparent increase in “mass,” however, may be misleading. Characterizations by Al-Ghadban et al. (2025) demonstrate that although skeletal muscle mass (SMM) appears to increase across lipedema stages, this increase does not translate into proportional functional capacity [5]. In contrast, van Esch-Smeenge et al. (2017) reported that women with lipedema exhibit markedly reduced quadriceps strength (~260–270 N) compared with BMI-matched obese controls (~400 N) [19].

This dissociation defines lipedematous dynapenia: impaired muscle strength despite preserved or increased tissue volume. Importantly, this phenomenon becomes clinically evident in Stage 3 but may biologically originate in Stage 2.5, where inflammatory burden, mechanical restriction, and metabolic inflexibility begin to converge.

We hypothesize that a substantial portion of this limb volume represents a form of pseudo-hypertrophy, composed not of functional myofibrillar expansion but of a disorganized matrix including intracellular edema, extracellular fibrosis, and ectopic lipid deposition. Support for this concept comes from sodium-MRI studies by Crescenzi et al., which revealed elevated sodium content in the skin and skeletal muscle of patients with lipedema, consistent with chronic low-grade inflammatory edema that expands tissue volume while compromising contractile efficiency [20]. In parallel, Shiose et al. demonstrated that muscle inflammation and injury significantly distort bioimpedance measurements, leading to systematic overestimation of muscle mass due to the conductive properties of interstitial fluid [21].

### 2.2. The Failure of Standard Metrics: Why BMI Is Blind

Failure to recognize this paradox is partly attributable to continued reliance on body mass index (BMI). As emphasized by Borga et al. (2018), BMI is inherently insensitive to body-composition quality and cannot differentiate functional myofibrillar tissue from pathological infiltration or edema [22]. In the context of lipedema, Taylor et al. (2022) employed chemical-shift–encoded MRI (Dixon technique) to demonstrate that muscle quality, assessed by fat fraction and radiodensity, is significantly compromised despite preserved limb size [23].

Accordingly, apparent preservation of limb circumference may mask a progressive degradation of muscle quality across advancing stages of lipedema. Limbs often described clinically as “strong” are, in fact, disproportionately heavy, metabolically inefficient, and burdened by non-contractile tissue. Recognition of this stage-dependent divergence between mass and function is central to understanding the functional inflection that characterizes the transition from Stage 2.5 to Stage 3.

### 2.3. Inflammatory Myosteatosis as a Metabolic Bottleneck

A central mechanism underlying this functional impairment is myosteatosis, defined as pathological accumulation of lipid within and between skeletal muscle fibers. In metabolic diseases such as obesity, insulin resistance, and type 2 diabetes, intramuscular lipid content correlates more strongly with functional limitation and metabolic dysfunction than muscle mass alone [24]. Experimental work by Gumucio et al. demonstrates that impaired mitochondrial lipid oxidation precedes intramyocellular fat accumulation following muscle injury or atrophy [25].

By analogy, we hypothesize that in lipedema, skeletal muscle mitochondria are exposed to excess free fatty acids but exhibit reduced metabolic flexibility and oxidative capacity. This mismatch may promote intramyocellular lipid accumulation, lipotoxic stress, and impaired contractile performance [24]. Within this framework, myosteatosis is positioned not as a passive consequence of inactivity, but as an active metabolic bottleneck that amplifies functional decline.

### 2.4. From Lipedema to Dynapenia: A Unified Mechanistic Axis

Lipedema is characterized by chronic adipose inflammation, extracellular fluid accumulation, microvascular dysfunction, and altered lipid handling. Evidence from aging and inflammatory cohorts indicates that elevated circulating cytokines such as IL-6 are independently associated with decline in muscle strength, even in the absence of proportional muscle mass loss [16]. These findings support the concept that inflammatory signaling may impair neuromuscular function beyond simple atrophy.

Within this context, inflammatory myosteatosis emerges as a plausible intermediate phenotype linking adipose expansion to contractile dysfunction. Longitudinal imaging studies demonstrate that intermuscular adipose tissue increases with aging and is associated with reductions in muscle strength and muscle quality independent of muscle cross-sectional area [17]. Importantly, muscle strength declines at a rate exceeding loss of muscle mass, reinforcing the dissociation between quantity and quality [15].

Experimental evidence further indicates that age-related reductions in excitation–contraction coupling efficiency contribute to loss of muscle force independent of muscle mass. Aging muscle exhibits reduced density of dihydropyridine receptors (DHPR) and impaired coupling to ryanodine receptors, resulting in diminished calcium release and compromised force generation [26]. Such excitation–contraction uncoupling provides a mechanistic basis for qualitative decline in muscle performance despite preserved muscle size. In addition, mitochondrial dysfunction and impaired oxidative capacity are recognized contributors to reduced muscle quality and contractile efficiency.

In parallel, short-term disuse has been shown to produce rapid reductions in muscle protein synthesis, lean mass, and strength in older adults [18], supporting the role of inactivity as an amplifying mechanism of muscle decline.

Together, interacting mechanisms including adipose-derived inflammatory signaling, ectopic lipid infiltration, mitochondrial inefficiency, excitation–contraction uncoupling, and disuse-related deconditioning create a biological scenario in which skeletal muscle mass may increase quantitatively while deteriorating qualitatively, producing a dynapenia-like phenotype despite preserved or even enlarged limb volume.

Dynapenia is inherently multifactorial and may arise from age-related motor unit remodeling and excitation–contraction uncoupling [15,26], metabolic disorders such as diabetes and obesity-associated myosteatosis [17], chronic systemic inflammation [16], endocrine contributors such as vitamin D deficiency and secondary hyperparathyroidism [27], reduced protein synthesis, and prolonged physical inactivity [18]. Lipedema may intersect with several of these pathways, particularly inflammation-driven mitochondrial dysfunction and inactivity-mediated deconditioning. However, unlike classical obesity-associated dynapenia, lipedema-related dysfunction may involve localized adipose–muscle inflammatory crosstalk combined with mechanical intolerance to loading.

Beyond its mechanical role, skeletal muscle functions as an endocrine organ capable of secreting a broad range of myokines that exert autocrine, paracrine, and endocrine effects [28]. These molecules, including IL-6, IL-15, irisin, myostatin, and SPARC, regulate systemic metabolism, adipose tissue remodeling, immune modulation, and mitochondrial function [29]. Exercise-induced IL-6, for example, exerts context-dependent anti-inflammatory and metabolic effects distinct from chronically elevated inflammatory IL-6 derived from immune sources [29]. IL-15 and irisin have been implicated in lipid oxidation, adipose browning, and enhancement of mitochondrial bioenergetics, reinforcing the bidirectional muscle–adipose communication axis [29]. Conversely, myostatin, an established negative regulator of muscle mass, promotes activation of SMAD2/3 signaling and catabolic pathways when dysregulated, contributing to impaired regeneration and anabolic resistance [28].

In the context of inflammatory myosteatosis, altered myokine secretion patterns or receptor-level signaling dysfunction may further disrupt adipose–muscle crosstalk, amplifying metabolic inflexibility and reducing adaptive responses to mechanical loading. Within this framework, myokine imbalance may represent an additional mechanistic layer linking adipose pathology to skeletal muscle dysfunction and dynapenia in advanced lipedema.

Beyond alterations in absolute myokine concentrations, qualitative dysregulation of secretion patterns may further contribute to impaired adipose–muscle crosstalk. Exercise-induced IL-6 is typically released in pulsatile bursts with context-dependent anti-inflammatory and metabolic effects, whereas chronically elevated IL-6 derived from immune sources reflects persistent inflammatory activation and is associated with muscle strength decline [16,30]. In advanced lipedema, sustained low-grade inflammation may blunt beneficial exercise-related myokine signaling while amplifying catabolic pathways. Additionally, extracellular matrix fibrosis and tissue stiffening described in lipedematous adipose depots [5] may impair local diffusion gradients and microvascular delivery of muscle-derived signaling molecules, potentially limiting effective paracrine communication. Emerging evidence also indicates that skeletal muscle releases extracellular vesicles and exosomal cargo that mediate inter-organ metabolic regulation [28]. Disruption of this vesicular signaling axis represents an additional, unexplored mechanism through which inflammatory myosteatosis could impair adaptive muscle–adipose communication, warranting targeted investigation.

**Figure 1:**
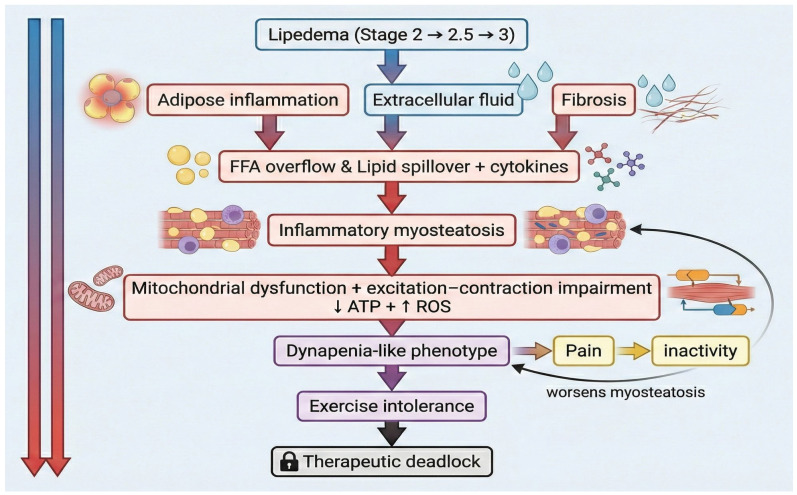
Mechanistic Axis of Lipedematous Dynapenic Myosteatosis. Schematic representation of the proposed pathophysiological bridge linking lipedema progression to dynapenia. Disease advancement from Stage 2 through Stage 2.5 to Stage 3 is associated with adipose inflammation, extracellular fluid accumulation, and fibrosis. These changes promote free fatty acid (FFA) overflow and cytokine signaling, contributing to inflammatory myosteatosis. Subsequent mitochondrial dysfunction and excitation–contraction impairment—characterized by reduced ATP availability and increased reactive oxygen species (ROS) production—lead to qualitative deterioration of muscle performance despite preserved or increased limb volume. This culminates in a dynapenia phenotype, exercise intolerance, and a therapeutic deadlock. Pain-driven inactivity further exacerbates myosteatosis, reinforcing a self-perpetuating cycle of functional decline. This figure was conceptualized and created by the authors using AI-assisted graphical design software (ChatGPT 5.2 and Gemini 3 Nano Banana), followed by manual editing and refinement. No copyrighted images or third-party graphical elements were used.

The integrated biological interactions described above are summarized schematically in Figure 1, illustrating the proposed mechanistic bridge between lipedema progression, inflammatory myosteatosis, and dynapenia. To anchor this conceptual model in empirical evidence, Table 1 provides a structured overview of the key translational and experimental studies supporting the proposed axis.

### 2.5. The Vicious Cycle of Pain, Inactivity, and Functional Decline

Building upon the mechanistic axis described above, the metabolic and structural disturbances of inflammatory myosteatosis have direct clinical and psychosocial consequences. Intramuscular lipid accumulation and chronic inflammatory signaling may sensitize nociceptors, contributing to the deep, aching pain reported by patients with lipedema. Observational data from Erbacher and Bertsch (2020) suggest that pain in lipedema extends beyond purely mechanical factors and is influenced by persistent inflammation and chronic disease-related distress [32]. Population-level data from Czech women with lipedema further demonstrate substantial impairments in physical and mental quality of life, underscoring the pervasive functional and psychosocial burden of the disease [33].

Pain-driven avoidance of movement promotes disuse atrophy and further mitochondrial deconditioning. Pain-related motor inhibition may additionally reduce effective muscle recruitment, compounding excitation–contraction inefficiency and limiting adaptive loading responses. Reduced oxidative capacity exacerbates myosteatosis, reinforcing a self-perpetuating cycle of pain, inactivity, and functional deterioration—a trajectory described by Aitzetmüller-Klietz et al. (2023) as a progressive loop of decline in advanced disease stages [34,35]. Within this framework, immobility emerges not as a behavioral failure, but as a predictable physiological consequence of intersecting inflammatory, metabolic, and neuromuscular dysfunction.

Taken together, the mechanisms described above indicate that functional impairment in advanced lipedema arises from a self-reinforcing interaction between metabolic inflexibility, inflammation, mitochondrial inefficiency, and declining muscle quality, limiting the effectiveness of conventional lifestyle-based interventions. The following section introduces a hypothesis-driven conceptual framework exploring how modulation of the metabolic milieu may alter the biological context in which functional recovery becomes feasible, without implying therapeutic efficacy.

## 3. The Therapeutic Deadlock and a Translational, Hypothesis-Driven Pharmacological Bypass

Building on the mechanisms described above, this section introduces a hypothesis-driven conceptual framework integrating metabolic inflexibility and skeletal muscle dysfunction in advanced lipedema. The aim is not to propose therapeutic interventions, but to clarify how tissue environment conditions the feasibility of exercise adaptation and functional recovery, without implying clinical efficacy.

### 3.1. The Exercise Paradox: Why Mechanical Loading May Fail

Resistance exercise is conventionally regarded as the cornerstone intervention for dynapenia and sarcopenia in healthy and metabolically stable populations. However, in lipedema, the physiological context in which mechanical loading occurs appears fundamentally altered. Emerging consensus statements from the Italian Society of Motor and Sports Sciences (SISMES) and the Italian Society of Phlebology (SIF) caution that high-intensity exercise may exacerbate inflammation, increase capillary filtration, and worsen edema in patients with lipedema, particularly in Stage 3 and severe functional phenotypes [36]. These effects are attributed to lactate accumulation, tissue hypoxia, and microvascular fragility within an already inflamed adipose–muscle interface.

In tissues characterized by chronic low-grade inflammation, contractile activity is known to modulate cytokine signaling and local immune responses in a context-dependent manner [30]. Moreover, high mechanical strain, particularly under conditions of metabolic stress, may amplify inflammatory cascades and interstitial fluid shifts, contributing to post-exercise edema and delayed functional recovery [37].

**Figure 2:**
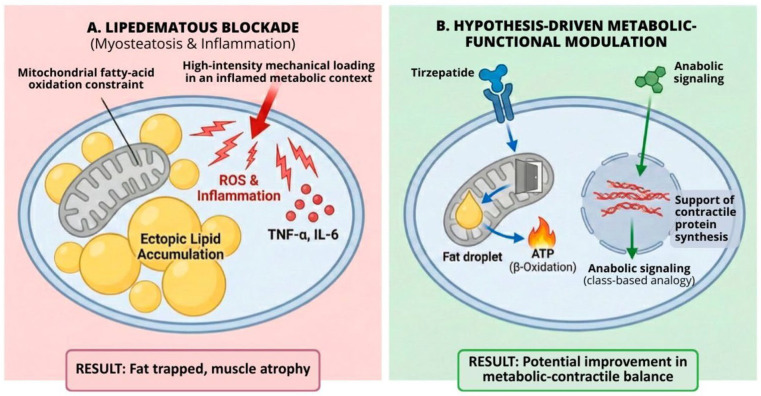
Conceptual model of lipedematous dynapenic myosteatosis and a hypothesis-driven pharmacological bypass. (**A**) Lipedematous blockade. In the untreated state, intramyocellular lipid accumulation (myosteatosis) is shown in association with mitochondrial inflexibility, illustrated as impaired CPT-1–mediated fatty acid transport. High-intensity mechanical loading may further amplify reactive oxygen species (ROS) production and pro-inflammatory cytokine signaling (e.g., TNF-α, IL-6), favoring muscle dysfunction and atrophy rather than adaptive hypertrophy. (**B**) Hypothesis-driven pharmacological bypass. A dual-target conceptual framework is illustrated. Metabolic modulation through dual GIP/GLP-1 receptor agonism (e.g., tirzepatide) may enhance mitochondrial substrate oxidation and reduce ectopic lipid accumulation. In parallel, anabolic signaling—shown using oxandrolone as an illustrative example—may theoretically promote contractile protein synthesis via nuclear androgen receptor pathways, partially reducing dependence on high mechanical loading. This figure was conceptualized and created by the authors using AI-assisted graphical design software (ChatGPT and Gemini), followed by manual editing and refinement. No copyrighted images or third-party graphical elements were used.

This clinical scenario gives rise to what we conceptualize as a therapeutic deadlock (Figure 2). On one hand, reversal of established dynapenia requires a sufficient anabolic stimulus. On the other, the mechanical stress typically required to induce such adaptation may trigger disproportionate inflammatory and edematous responses. As reported by Aitzetmüller-Klietz et al. [34] and Annunziata et al. [6,21,32,36,38,39,40], high-intensity loading in lipedema frequently aggravates pain and swelling, leading to exercise intolerance and subsequent avoidance. The patient thus becomes biologically constrained: metabolically limited by impaired lipid oxidation and mechanically limited by inflammatory hypersensitivity, rendering conventional “train harder” strategies physiologically impractical rather than behaviorally deficient.

### 3.2. The Dual-Target Hypothesis: Breaking the Pathophysiological Cycle

To address the therapeutic deadlock observed in advanced lipedema, we propose a dual-target, hypothesis-driven framework that considers metabolic inflexibility and contractile dysfunction as interdependent, but hierarchically organized, components of functional decline. Rather than implying a direct pharmacological shortcut or a linear intervention–response sequence, this framework seeks to interrupt the self-reinforcing biological loops that sustain inflammatory myosteatosis. These loops contribute to exercise intolerance and progressive loss of mobility in lipedema.

Within this model, metabolic dysregulation is positioned as the primary enabling constraint. It shapes the tissue environment in which mechanical loading and muscle adaptation occur. Contractile dysfunction is therefore conceptualized not as an isolated muscular defect, but as a downstream consequence of chronic inflammation, ectopic lipid accumulation, and impaired mitochondrial substrate handling. This distinction is critical, as it implies that restoration of muscle function cannot be achieved through mechanical or anabolic stimuli alone unless the underlying metabolic milieu is first reconditioned.

#### 3.2.1. Metabolic Modulation: Tirzepatide and Mitochondrial Flexibility

Restoration of mitochondrial lipid handling and oxidative flexibility represents a biological prerequisite for mitigating inflammatory myosteatosis and improving muscle quality in lipedema. As discussed in detail in our previous narrative review on tirzepatide in lipedema, mitochondrial dysfunction, impaired fatty acid oxidation, chronic inflammation, and extracellular matrix fibrosis constitute interrelated pathogenic nodes that sustain metabolic rigidity in lipedematous adipose depots and adjacent tissues [6,21,32,36,38,39,40].

Dual GIP/GLP-1 receptor agonism offers mechanistic advantages over GLP-1–only therapies by modulating lipid metabolism, immune signaling, and stromal remodeling beyond appetite suppression alone [22,23,32]. Experimental and translational evidence indicates that co-activation of GIP and GLP-1 receptors enhances mitochondrial efficiency, improves substrate utilization, and promotes adipose tissue remodeling, including increased thermogenic signaling and beige adipocyte differentiation [32,39,40]. Importantly, these effects are consistent with a global restoration of metabolic flexibility, rather than activation or inhibition of any single molecular pathway.

These mechanisms are particularly relevant to lipedema, where adipose tissue fibrosis, microvascular dysfunction, and mitochondrial inflexibility contribute to resistance to lipid mobilization and perpetuate ectopic lipid deposition in skeletal muscle [5,36,38]. In this context, tirzepatide may indirectly recondition the metabolic and inflammatory microenvironment surrounding myocytes by attenuating inflammatory signaling, reducing profibrotic pathways, and improving insulin sensitivity, thereby lowering lipid spillover into muscle fibers [21,32,39]. Tirzepatide is therefore conceptualized here as a metabolic enabler, restoring the oxidative capacity required to safely process mobilized substrates rather than acting directly on contractile tissue.

##### Lipolysis–Oxidation Mismatch

Isolated stimulation of lipolysis in the absence of adequate mitochondrial oxidative capacity is insufficient and may be metabolically maladaptive. Fatty acids mobilized without parallel enhancement of mitochondrial oxidation are prone to re-esterification or conversion into lipotoxic intermediates. This process exacerbates oxidative stress, mitochondrial dysfunction, and intramyocellular lipid accumulation [6,32,39]. This lipolysis–oxidation mismatch has been identified as a key contributor to myosteatosis and impaired muscle performance in chronic metabolic and inflammatory states.

Within this framework, signaling pathways that promote lipid mobilization or nitrogen retention may increase substrate availability but do not, in isolation, resolve ectopic lipid overload. Without concurrent restoration of mitochondrial flexibility, such mechanisms risk amplifying lipid overflow rather than alleviating it [32,36,38]. Consequently, mitochondrial reactivation through metabolic modulation is foundational, providing the oxidative capacity necessary to dissipate mobilized substrates and prevent further ectopic lipid deposition. This hierarchy underscores why metabolic modulation must precede, or at least accompany, any strategy aimed at restoring muscle contractile function.

Once the metabolic and inflammatory milieu is partially reconditioned, the biological context in which mechanical loading occurs is fundamentally altered. In the absence of such reconditioning, higher-intensity resistance exercise applied to a rigid, inflamed, and metabolically inflexible tissue environment is more likely to propagate oxidative stress, pain, and edema rather than induce adaptive hypertrophy.

This helps explain why conventional training strategies may fail or even exacerbate symptoms in advanced lipedema, not because mechanical loading is intrinsically ineffective, but because it is introduced in a biologically unfavorable phase of the disease. Within this framework, restoration of metabolic flexibility is expected to reduce inflammatory amplification during exercise, thereby lowering the physiological cost of movement and permitting re-engagement with mechanical stimuli under more tolerable conditions.

Only in this reconditioned context does it become conceptually appropriate to discuss functional support mechanisms aimed at restoring strength and contractile capacity, which are addressed below as a secondary and dependent component of the proposed framework.

#### 3.2.2. Anabolic Signaling as a Functional Rescue Analogy

In this reconditioned metabolic context, where inflammatory burden and mitochondrial inflexibility are at least partially attenuated, limitations in skeletal muscle function become more clearly interpretable as a downstream consequence of prior chronic catabolic stress rather than as an immutable structural defect. In advanced stages of lipedema (Stage 3), skeletal muscle may exhibit features consistent with localized, sustained catabolism. These features include reduced strength, impaired mobility, and diminished tolerance to mechanical loading. Earlier stages (Stage 2 and transitional phenotypes) should therefore be regarded as a critical window for proactive functional assessment, as overt disability is most consistently recognized only once Stage 3 is established.

Within this framework, it is informative to draw a mechanistic analogy with established catabolic conditions, such as HIV-associated wasting, severe burns, trauma, and chronic inflammatory disease, in which augmentation of anabolic signaling has historically been explored as a means of preserving or restoring muscle function. Importantly, this analogy is used here to contextualize functional recovery, not to propose therapeutic transfer across disease states.

The discussion of anabolic androgenic steroids (AAS) in this manuscript is strictly confined to a functional and rehabilitative framework. The objective is the preservation or recovery of contractile capacity and mobility under conditions of documented catabolic stress, not aesthetic modification, performance enhancement, or therapeutic recommendation. Accordingly, AAS are referenced as a pharmacological class, serving as an illustrative model for how anabolic signaling may influence muscle function when biological prerequisites for adaptation are met.

Oxandrolone is cited as a representative example within this class, given its extensive documentation in the literature as an adjunct therapy in conditions characterized by severe catabolic imbalance, sarcopenia, and dynapenia. In HIV-associated wasting, oxandrolone has been shown to improve lean body mass, nitrogen balance, and functional parameters, including in women, under controlled clinical conditions [41,42]. When combined with structured exercise interventions, anabolic strategies in HIV populations further demonstrated improvements in muscle performance and functional outcomes, reinforcing the concept that anabolic signaling requires adequate mechanical and nutritional support to translate into benefit [43].

In pediatric populations exposed to profound catabolic stress, including malnourished children with HIV and severe burn injury, oxandrolone has likewise been investigated as a rehabilitative adjunct. Clinical studies and systematic reviews report improvements in lean mass accretion, growth parameters, and functional recovery when oxandrolone is used within carefully monitored protocols [44,45]. These data underscore that the primary rationale for oxandrolone use in such contexts is functional recovery, rather than cosmetic or performance-related outcomes.

Importantly, contemporary systematic reviews evaluating oxandrolone across catabolic and chronic disease states conclude that its effects on muscle mass and function are context-dependent, modest in magnitude, and contingent upon appropriate nutritional intake, metabolic support, and clinical monitoring [46]. These analyses further emphasize that anabolic signaling alone does not override energetic constraints and should not be interpreted as a standalone solution for muscle dysfunction [29].

From a mechanistic perspective, anabolic signaling may favor nutrient partitioning toward skeletal muscle. It may enhance protein synthesis efficiency while limiting excessive proteolysis. However, effective muscle protein synthesis requires sufficient caloric intake, adequate protein availability, and an intact oxidative environment. Severe caloric restriction or persistent metabolic inflexibility would be expected to blunt anabolic responsiveness regardless of signaling intensity.

Within the proposed hypothesis-driven framework, metabolic modulation remains foundational, establishing the energetic and oxidative conditions necessary for anabolic signaling to translate into functional benefit. In this sequence, anabolic signaling, illustrated here by oxandrolone and related agents, should be interpreted as secondary and dependent, potentially facilitating recovery of strength and tolerance to mechanical loading once the metabolic–inflammatory milieu has been favorably modified. This hierarchy reinforces that restoration of muscle function in complex metabolic diseases such as lipedema cannot be achieved through isolated anabolic stimulation, but rather through coordinated modulation of metabolic environment, nutritional adequacy, and mechanical exposure.

In summary, this hypothesis-driven framework positions dynapenia in advanced lipedema as a context-dependent outcome of metabolic and inflammatory constraints rather than an isolated muscular defect. The model is non-prescriptive and does not infer clinical efficacy but serves to generate mechanistically informed hypotheses to be tested in future translational and clinical studies.

## 4. Discussion: Translational Implications and Safety Considerations

### 4.1. Identifying the Functional Inflection Point

The pharmacological framework discussed in this manuscript is not intended for early-stage lipedema, in which conservative strategies such as compression therapy, lymphatic management, and low-impact physical activity remain appropriate. Rather, the hypothesis is oriented toward a functional inflection point, at which progressive tissue expansion begins to compromise biomechanics, muscle quality, and mobility.

This perspective is consistent with recent disease re-characterization showing that skeletal muscle mass, interstitial fluid accumulation, and functional performance represent partially independent dimensions of disease severity that do not correlate linearly with BMI [5]. Within this framework, overt functional impairment is most consistently recognized in Stage 3; however, the transition from Stage 2 to Stage 2.5 may represent a biologically critical window in which early functional signals emerge.

**Figure 3:**
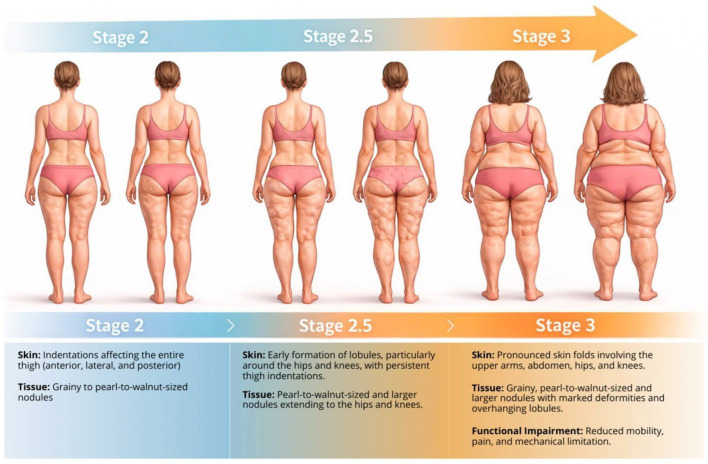
Morphological progression of lipedema from Stage 2 to Stage 3 and its relationship with functional impairment. Representative schematic illustration of lipedema Stages 2, 2.5, and 3, highlighting progressive morphological changes in skin and subcutaneous tissue. Stage 2 shows diffuse thigh indentations with grainy to pearl-to-walnut-sized nodules; Stage 2.5 represents a transitional phenotype with early lobule formation around the hips and knees; Stage 3 is characterized by pronounced skin folds, large deforming lobules, and clinically evident functional impairment, including reduced mobility, pain, and mechanical limitation. The figure preserves established morphological staging and emphasizes that functional impairment is most consistently recognized at Stage 3, while earlier stages may warrant proactive functional assessment. The illustration is intended for conceptual clarification and does not redefine diagnostic or staging criteria. It was conceptualized and created by the authors using AI-assisted graphical design software (ChatGPT and Gemini), followed by manual editing and refinement. No copyrighted images or third-party graphical elements were used.

The principal clinical challenge is not the identification of advanced disease. Rather, it is the timely recognition of transitional phenotypes in which dynapenic features begin to manifest. Early functional assessment, including objective strength testing and performance-based measures, may allow intervention before mechanical restriction and inflammatory amplification become entrenched. Figure 3 contextualizes this morphological-to-functional progression without redefining established staging criteria.

Rather than serving as diagnostic categories, these images are intended to contextualize a spectrum in which mechanical restriction, inflammatory burden, and muscle dysfunction progressively converge. At this stage, pharmacological strategies are discussed solely as a conceptual research consideration in individuals who demonstrate objective evidence of functional loss, inflammatory mechanical intolerance, and refractoriness to conservative management. Table 2 therefore represents a hypothesis-generating clinical phenotype, not a treatment algorithm. Importantly, the domains outlined below are intended for research stratification and hypothesis generation only, and should not be interpreted as criteria for clinical decision-making.

### 4.2. Contextualizing the Anabolic Rationale Within Evidence-Based Medicine

Oxandrolone occupies a distinct position among AAS due to its relatively low androgenicity and extensive historical use in catabolic conditions. Seminal work by Orr and Fiatarone Singh [38] and others has documented its use in severe burn injury, trauma, and chronic wasting states, where preservation of lean mass and bone density was achieved with acceptable long-term safety profiles. These populations represent extreme models of sustained catabolism rather than aesthetic or performance contexts.

Importantly, data from these settings are not invoked to justify direct clinical transfer. Instead, they support mechanistic plausibility. Lipedematous skeletal muscle in advanced disease may share features with chronically catabolic tissue. From this perspective, anabolic signaling may theoretically function as a contractile rescue mechanism, rather than as a driver of hypertrophy per se.

### 4.3. Safety Considerations in a Hypothesis-Driven Framework

Any translational consideration of anabolic signaling in lipedema must be accompanied by explicit safety acknowledgment. Oxandrolone, as a 17-α-alkylated steroid, carries a recognized hepatic signal, necessitating careful contextualization. In therapeutic catabolic models, transient aminotransferase elevations were common but typically reversible, with clinically significant hepatotoxicity remaining rare under controlled conditions [38,47].

Beyond hepatic considerations, oral AAS are associated with reductions in HDL cholesterol, raising theoretical cardiovascular concerns in long-term use. In the conceptual framework proposed here, concurrent metabolic modulation via incretin-based therapy introduces a potential counterbalance. Dual GLP-1/GIP receptor agonists have consistently demonstrated favorable effects on triglycerides, LDL-cholesterol, and systemic inflammation [48], suggesting a theoretical interaction that warrants cautious evaluation, rather than presuming additive cardiometabolic risk.

Fluid handling represents an additional consideration. Androgen-mediated sodium retention could theoretically exacerbate edema in lipedema. However, GLP-1 receptor signaling has been associated with natriuresis and improved endothelial function. While such interactions remain speculative, they underscore the necessity of integrated metabolic-hormonal reasoning rather than isolated pharmacological thinking. While biologically plausible, this framework should be regarded strictly as hypothesis-generating. It is intended to organize emerging translational insights within a coherent adipose–muscle model and to stimulate mechanistically informed clinical investigation, rather than to imply clinical efficacy or guide therapeutic sequencing.

### 4.4. Non-Pharmacological Strategies Within the Proposed Framework

Non-pharmacological strategies remain the cornerstone of lipedema management across all stages of disease progression. Conservative interventions, including compression therapy, lymphatic management, structured physical activity, and individualized dietary strategies, continue to represent first-line approaches and are supported by consensus recommendations and clinical practice guidelines [13,36]. Within this context, the hypothesis-driven framework proposed in the present review does not seek to replace conservative care, but rather to contextualize its variable efficacy in advanced disease stages characterized by inflammatory myosteatosis and dynapenic features.

Emerging consensus statements from the Italian Society of Motor and Sports Sciences (SISMES) and the Italian Society of Phlebology (SIF) emphasize the therapeutic role of structured, low-impact resistance training, aquatic exercise, and progressive functional conditioning in individuals with lipedema [36]. These modalities aim to improve muscle strength, mobility, and metabolic health while minimizing inflammatory exacerbation and post-exertional edema. Clinical observations and patient-reported outcomes suggest that individualized exercise prescriptions, particularly those incorporating gradual load progression and symptom monitoring, may improve functional capacity in earlier stages of lipedema (Stages 1–2) and in transitional phenotypes (Stage 2.5). Importantly, however, exercise tolerance appears stage-dependent, with advanced Stage 3 patients frequently reporting mechanical intolerance, persistent post-exertional pain, and edema exacerbation [34].

From a pathophysiological standpoint, these observations align with the proposed adipose–muscle axis described in earlier sections. In early and metabolically stable stages, resistance exercise likely enhances mitochondrial function, improves substrate utilization, and stimulates favorable myokine secretion patterns, thereby supporting muscle–adipose crosstalk. Conversely, in advanced stages characterized by chronic inflammation, impaired lipid mobilization, extracellular matrix remodeling, and microvascular dysfunction [5,20], mechanical loading may occur within a metabolically inflexible and inflammatory milieu. Under such conditions, the physiological cost of exercise may exceed adaptive capacity. This imbalance may contribute to the “exercise paradox” previously described. Dietary strategies further modulate this interaction. While lipedema is distinct from primary obesity, excess adiposity may exacerbate inflammatory burden and mechanical load [4,7]. Nutritional approaches aimed at improving insulin sensitivity, reducing systemic inflammation, and optimizing protein intake may therefore indirectly support muscle quality and functional performance. Importantly, the current literature does not support extreme caloric restriction as a disease-modifying strategy in lipedema, particularly in advanced stages where resistance to fat mobilization and psychosocial distress are prevalent [6,11].

Within the integrated framework proposed in this review, non-pharmacological strategies should be viewed as foundational and stage-sensitive. Conservative management remains the primary therapeutic axis, particularly in Stages 1–2.5, where structured resistance training and dietary modulation may prevent or attenuate progression toward dynapenic phenotypes. The pharmacological considerations discussed in Section 3 are presented solely as hypothesis-generating adjuncts for investigational contexts involving objectively documented functional decline and refractoriness to conservative management. Accordingly, the proposed model emphasizes biological sequencing rather than therapeutic substitution: metabolic stability, functional conditioning, and individualized load tolerance remain central to clinical care across the lipedema spectrum.

### 4.5. Methodological Gaps and Future Research Priorities

Despite the conceptual integration proposed in this review, direct empirical validation of the lipedema–inflammatory myosteatosis–dynapenia axis remains limited. Several critical methodological gaps must be addressed before the framework can be substantiated or refuted.

First, there is a complete absence of skeletal muscle biopsy studies in individuals with lipedema. Histological characterization of intramyocellular lipid content, mitochondrial density, fiber-type distribution, inflammatory infiltration, and extracellular matrix remodeling would provide essential mechanistic evidence. Without direct tissue-level analysis, current inferences rely on extrapolation from metabolic and aging cohorts. This represents one of the most critical limitations of the proposed framework.

Second, longitudinal imaging studies evaluating muscle quality across lipedema stages are lacking. While cross-sectional MRI and CT data suggest altered tissue composition, no prospective studies have tracked muscle fat fraction, radiodensity, or structural remodeling during progression from Stage 2 to Stage 3. Establishing temporal relationships between adipose expansion, inflammatory burden, and declining muscle performance is therefore a research priority.

Third, combined interventional studies integrating structured exercise protocols with advanced imaging modalities are absent. Trials incorporating resistance training alongside MRI-based fat fraction analysis, muscle radiodensity assessment, and objective strength testing could clarify whether improvements in muscle function correspond to measurable reductions in myosteatosis or shifts in tissue quality.

Fourth, the proposed lipolysis–oxidation mismatch hypothesis requires metabolic phenotyping beyond anthropometric or BMI-based measures. Simultaneous assessment of indirect calorimetry-derived respiratory quotient (RQ), insulin sensitivity indices, inflammatory markers, and imaging-based muscle quality would enable integrated evaluation of metabolic flexibility and structural adaptation. Such multimodal designs could determine whether impaired substrate oxidation precedes or follows structural muscle changes.

Finally, future pilot studies should be stratified by lipedema stage, particularly distinguishing Stage 2, Stage 2.5, and Stage 3 phenotypes. Stage-specific stratification is essential to determine whether inflammatory myosteatosis represents a late-stage consequence or an early mechanistic driver of functional decline. Without staging-based analysis, heterogeneity may obscure biologically meaningful patterns.

To facilitate structured investigation, Table 2 outlines a hypothesis-generating functional phenotype designed to operationalize the proposed adipose–muscle framework. The domains described are not intended as diagnostic or therapeutic criteria, but rather as research stratification tools integrating functional testing, imaging-based muscle quality assessment, and metabolic phenotyping. By combining objective dynapenia measures with structural and metabolic markers, this schema aims to support stage-specific study design and reduce heterogeneity in future translational trials.

**Table 2:** Hypothesis-generating functional phenotype for research stratification in advanced lipedematous dynapenia. Proposed domains, operational definitions (with literature-based examples), and objective endpoints to enable standardized phenotyping across lipedema stages. Intended for research stratification and hypothesis testing only.

Domain	Operational Definition & Assessment (Literature-Based Examples)	Pathophysiological Significance	Suggested Objective Endpoints
1. Objective dynapenia (function)	Timed Up and Go (TUG) > 10 s based on mobility risk thresholds [49,50,51]. Sit-to-Stand (5 × STS or 30 s STS) below age- and sex-adjusted normative values [52,53]. Handgrip strength below established cutoffs for sarcopenia/dynapenia [50,54,55].	Dissociation between limb volume and contractile performance, indicating failure of muscle quality rather than simple mass loss.	TUG, STS, handgrip strength, isokinetic quadriceps torque, gait speed, mobility-related patient-reported outcomes.
2. Mechanical intolerance and refractoriness to loading	Post-exertional pain persisting >24 h; measurable limb volume increase within 24–48 h after activity; persistence of symptoms despite ≥6 months of documented conservative therapy (compression, physiotherapy, diet) [56,57].	Captures the “exercise paradox,” in which inflammatory and edematous responses limit tolerance to mechanical loading and promote disuse atrophy.	Pain NRS/VAS, post-exertional symptom diary, limb volume change, pressure pain thresholds, accelerometry-based activity metrics.
3. Myosteatosis and impaired muscle quality (structure)	MRI Dixon fat fraction, muscle radiodensity (CT), or ultrasound echo-intensity based on established muscle quality imaging standards [17,31,58]	Links dynapenia to ectopic lipid deposition, inflammatory muscle remodeling, and reduced contractile efficiency.	Muscle fat fraction, radiodensity/echo-intensity, correlation with strength and mobility, longitudinal structural change.
4. Metabolic–inflammatory milieu consistent with oxidative mismatch	Insulin resistance indices, inflammatory cytokines, adipokines, fibrosis/ECM-related biomarkers, indirect calorimetry-derived RQ for metabolic flexibility [59,60].	Tests the proposed lipolysis–oxidation mismatch and immunometabolic drivers sustaining myosteatosis and functional decline.	RQ (respiratory quotient), insulin sensitivity, inflammatory panels, fibrosis markers, association with imaging and function.

Addressing these gaps will require interdisciplinary collaboration combining clinical phenotyping, advanced imaging, metabolic assessment, and mechanistic laboratory investigation. The proposed framework should therefore be interpreted as a scaffold for structured hypothesis testing rather than as a completed explanatory model.

## 5. Conclusions

This review reframes advanced lipedema not solely as a disorder of disproportionate adipose accumulation, but as a condition in which adipose inflammation, ectopic lipid deposition, and skeletal muscle bioenergetic dysfunction converge. We propose that inflammatory myosteatosis serves as a mechanistic bridge linking lipedema progression, particularly from Stage 2.5 to Stage 3, to a dynapenic phenotype characterized by qualitative deterioration of muscle performance despite preserved or increased limb volume.

Within this framework, functional decline emerges from the interaction between adipose-derived inflammatory signaling, mitochondrial inefficiency, excitation–contraction impairment, and disuse-related deconditioning. This integrated axis may help explain the dissociation between tissue mass and strength, the high prevalence of exercise intolerance in advanced stages, and the limited effectiveness of strategies focused exclusively on adipose reduction.

The dual-target concept discussed herein, combining metabolic reconditioning with support of contractile function, is presented strictly as a hypothesis-generating framework anchored in the proposed adipose–muscle axis. Its purpose is not to recommend pharmacological intervention, but to stimulate mechanistically informed clinical investigation.

Future studies should prospectively evaluate muscle quality, mitochondrial function, inflammatory signaling, and objective measures of strength and mobility across lipedema stages, particularly during the transition from Stage 2.5 to Stage 3. By shifting attention from adipose volume alone to the integrated triad of lipedema, inflammatory myosteatosis, and dynapenia, this framework provides a testable model for understanding functional decline in advanced disease.

## Figures and Tables

**Table 1:** Key Evidence Supporting the Lipedema–Inflammatory Myosteatosis–Dynapenia Axis. This table summarizes selected foundational studies that underpin the mechanistic framework proposed in this review. Included studies represent conceptual, longitudinal, experimental, and imaging-based evidence linking inflammation, intermuscular adipose infiltration, excitation–contraction impairment, disuse, and metabolic dysfunction to qualitative muscle decline. While not specific to lipedema in all cases, these investigations provide translational support for the hypothesized adipose–muscle bioenergetic axis described herein.

Study	Study Design (N)	Population	Methods	Key Findings	Relevance to Proposed Framework
Delbono, 2000 [26]	Mechanistic review (N/A)	Aging skeletal muscle (animal & human data)	Analysis of excitation–contraction coupling mechanisms	Reduced DHPR density and impaired coupling to RyR1 lead to decreased Ca^2+^ release and force	Supports excitation–contraction uncoupling as qualitative contributor to dynapenia
Goodpaster et al., 2000 [31]	Cross-sectional imaging study (n ≈ 70–80)	Older adults	CT muscle attenuation as proxy of intramuscular lipid content	Lower muscle attenuation associated with higher intramuscular lipid and reduced strength	Validates radiodensity as marker of muscle quality
Schaap et al., 2006 [16]	Prospective cohort (n = 986)	Community-dwelling older adults	Baseline IL-6, CRP, ACT; grip strength measured over 3 years	Elevated IL-6 and CRP associated with 2–3× increased risk of >40% decline in muscle strength	Supports inflammation-driven component of dynapenia
Kortebein et al., 2007 [18]	Experimental interventional study (n = 12; 10 analyzed for synthesis)	Healthy older adults (mean age 67 years)	10-day strict bed rest; muscle protein synthesis (FSR), DEXA lean mass, knee extension strength	−30% muscle protein synthesis; −6% lower extremity lean mass; −15.6% strength	Supports disuse-mediated dynapenia and rapid functional decline
Clark & Manini, 2008 [15]	Conceptual review (N/A)	Older adults	Narrative review of longitudinal and mechanistic studies	Muscle strength declines exceed loss of muscle mass; neural and excitation–contraction mechanisms contribute to dynapenia	Establishes the conceptual distinction between sarcopenia and dynapenia; supports the quality-over-quantity paradigm
Delmonico et al., 2009 [17]	Longitudinal cohort (n = 1678)	Older men and women	CT mid-thigh cross-sectional area (CSA), intermuscular fat (IMF), strength over 5 years	Strength declined 2–5× more than CSA; IMF increased with aging and correlated with reduced muscle quality	Supports dissociation between muscle mass and strength; age-related myosteatosis

## Data Availability

No new data were created or analyzed in this study. Data sharing is not applicable to this article.

## Abbreviations

30 s STS	30-Second Sit-to-Stand Test
5 × STS	Five Times Sit-to-Stand Test
AAS	Anabolic Androgenic Steroids
ACT	α1-Antichymotrypsin
ATP	Adenosine Triphosphate
BIA	Bioelectrical Impedance Analysis
BMI	Body Mass Index
Ca^2+^	Calcium Ion
CPT-1	Carnitine Palmitoyltransferase 1
CRP	C-Reactive Protein
CSA	Cross-Sectional Area
DEXA	Dual-Energy X-ray Absorptiometry
DHPR	Dihydropyridine Receptor
ECM	Extracellular Matrix
ERα	Estrogen Receptor Alpha
ERβ	Estrogen Receptor Beta
FFA	Free Fatty Acids
FFM	Fat-Free Mass
FSR	Fractional Synthesis Rate
GIP	Glucose-Dependent Insulinotropic Polypeptide
GLP-1	Glucagon-Like Peptide-1
HDL	High-Density Lipoprotein
HFpEF	Heart Failure with Preserved Ejection Fraction
HIV	Human Immunodeficiency Virus
IL-15	Interleukin-15
IL-6	Interleukin-6
IMF	Intermuscular Fat
LDL	Low-Density Lipoprotein
MRI	Magnetic Resonance Imaging
NRS	Numeric Rating Scale
ROS	Reactive Oxygen Species
RQ	Respiratory Quotient
RyR1	Ryanodine Receptor Type 1
SAT	Subcutaneous Adipose Tissue
SIF	Italian Society of Phlebology
SISMES	Italian Society of Motor and Sports Sciences
SMAD2/3	Mothers Against Decapentaplegic Homolog 2/3 (intracellular signaling proteins)
SMM	Skeletal Muscle Mass
SPARC	Secreted Protein Acidic and Rich in Cysteine
STS	Sit-to-Stand Test
TNF-α	Tumor Necrosis Factor Alpha
TUG	Timed Up and Go Test
VAS	Visual Analog Scale
